# Area-Specific Cell Stimulation via Surface-Mediated Gene Transfer Using Apatite-Based Composite Layers

**DOI:** 10.3390/ijms16048294

**Published:** 2015-04-14

**Authors:** Yushin Yazaki, Ayako Oyane, Yu Sogo, Atsuo Ito, Atsushi Yamazaki, Hideo Tsurushima

**Affiliations:** 1 Department of Resources and Environmental Engineering, Waseda University, 3-4-1 Okubo, Shinjuku, Tokyo 169-8555, Japan; E-Mails: yushin_0822@yahoo.co.jp (Y.Y.); ya81349@waseda.jp (A.Y.); 2 Nanomaterials Research Institute, National Institute of Advanced Industrial Science and Technology, Central 4, 1-1-1 Higashi, Tsukuba, Ibaraki 305-8562, Japan; 3 Health Research Institute, National Institute of Advanced Industrial Science and Technology, Central 6, 1-1-1 Higashi, Tsukuba, Ibaraki 305-8566, Japan; E-Mails: yu-sogou@aist.go.jp (Y.S.); atsuo-ito@aist.go.jp (A.I.); 4 Department of Neurosurgery, Faculty of Medicine, University of Tsukuba, 1-1-1 Tennoudai, Tsukuba, Ibaraki 305-8575, Japan; E-Mail: hideo-tsurushima@md.tsukuba.ac.jp

**Keywords:** dual gene transfer, hydroxyapatite, reverse transfection, calcium phosphate, scaffold

## Abstract

Surface-mediated gene transfer systems using biocompatible calcium phosphate (CaP)-based composite layers have attracted attention as a tool for controlling cell behaviors. In the present study we aimed to demonstrate the potential of CaP-based composite layers to mediate area-specific dual gene transfer and to stimulate cells on an area-by-area basis in the same well. For this purpose we prepared two pairs of DNA–fibronectin–apatite composite (DF-Ap) layers using a pair of reporter genes and pair of differentiation factor genes. The results of the area-specific dual gene transfer successfully demonstrated that the cells cultured on a pair of DF-Ap layers that were adjacently placed in the same well showed specific gene expression patterns depending on the gene that was immobilized in theunderlying layer. Moreover, preliminary real-time PCR results indicated that multipotential C3H10T1/2 cells may have a potential to change into different types of cells depending on the differentiation factor gene that was immobilized in the underlying layer, even in the same well. Because DF-Ap layers have a potential to mediate area-specific cell stimulation on their surfaces, they could be useful in tissue engineering applications.

## 1. Introduction

Safe, efficient, and area-specific gene transfer systems for controlling cell behaviors, including proliferation and differentiation, are useful tools for tissue engineering. Conventional nonviral gene transfer systems are generally mediated by particulate complexes of DNA and transfection reagents, such as lipids [1,2,3], cationic polymers [4,5,6,7], and calcium phosphates (CaPs) [8,9,10,11,12,13]. These particle-mediated systems are usually weak in area-specific gene transfer and are incapable of stimulating cells at a specific location. This is because particulate DNA complexes easily disperse in a culture medium or in body fluids.

Since the late 1990s, surface-mediated gene transfer systems (also called reverse transfection) that are capable of area-specific gene transfer have been developed [14,15,16,17,18,19,20,21,22,23,24,25,26,27,28,29,30,31,32]. In these systems, the cells are seeded onto substrates with immobilized DNA and, in many cases, with transfection reagents as well. The immobilized DNA is released from the substrate and then taken up by the cells that have adhered to the substrate. Recently, other approaches that use physical stimulations, including electric pulses [33,34,35], ultrasound [36,37,38], and atmospheric plasmas [39,40,41], have also been proposed for achieving area-specific gene transfer.

Among such surface-mediated gene transfer systems, we have focused on a system that uses CaP-based composite layers [18,19,20,21,22,23,24,25,26,27] toward *in vitro* and *in vivo* tissue engineering applications. CaP-based composite layers consist of a matrix of CaP with immobilized DNA and can be coated onto a variety of scaffold materials [25]. Owing to the good biocompatibility of CaP, these composite layers support cell viability on their surfaces with minimal toxicity [18]. Furthermore, these composite layers can be designed to exhibit increased gene transfer efficiency by coimmobilization of biofunctional molecules such as cell adhesion molecules (laminin [19,20], fibronectin [21,22]), and lipids [23,24,26,27] within the composite layer. We have demonstrated that CaP-based composite layers have the potential to accelerate not only *in vitro* cell differentiation [20,42,43], but also *in vivo* bone tissue regeneration [42] on the layers. Our preliminary studies showed that a DNA–fibronectin–apatite composite layer (DF-Ap layer) potentially allow area-specific gene transfer on their surfaces using a simple assay system based on a luciferase reporter gene [21]. Area-specific gene transfer was suggested by the observation that the cells cultured on the composite layer shows significantly higher luciferase activity than the cells cultured on the well around the composite layer in the same well [21].

In the present study, we aimed to demonstrate the potential of CaP-based composite layers to mediate area-specific dual gene transfer and cell stimulation in the same well. For this purpose, we designed two experimental systems using two pairs of DF-Ap layers: a pair of layers immobilizing reporter genes for area-specific dual gene transfer study and pair of layers immobilizing differentiation factor genes for area-specific cell stimulation (Table 1).

**Table 1:** List of the genes, plasmid DNAs, sample names, and cell lines used in this study.

Type of Gene	Gene	Plasmid DNA	Sample Name	Transfected Cell
Reporter gene	*Firefly luciferase* (*FL*)	pGL3 ^(1)^	DF-FL	CHO-K1 cell
	*Renilla luciferase* (*RL*)	pRL-TK ^(2)^	DF-RL	
Differentiation factor gene	*Vascular endothelial growth factor* (*VEGF*)	pCI-VEGF ^(3)^	DF-V	C3H10T1/2 embryonic cell
	*Bone morphogenetic protein-2* (*BMP-2*)	pCI-BMP ^(4)^	DF-B	

^(1)^ pGL3: pGL3-Control vector including the cDNA of *firefly luciferase* (*FL*); ^(2)^ pRL-TK: pRL-TK vector including the cDNA of *Renilla luciferase* (*RL*); ^(3)^ pCI-VEGF: pCI-neo vector including the cDNA of human *vascular endothelial growth factor* (*VEGF*); ^(4)^ pCI-BMP: pCI-neo vector including the cDNA of human *bone morphogenetic protein-2* (*BMP-2*).

**Figure 1:**
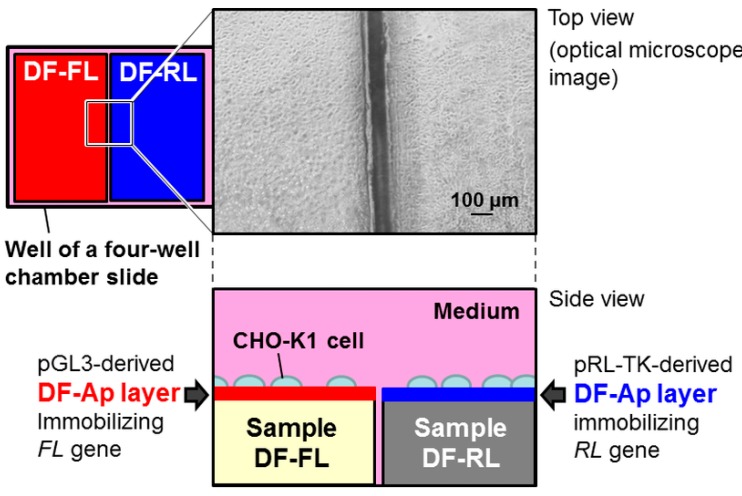
Schematic depiction of the sample setting used in the dual reporter gene transfer study.

First, we prepared two types of DF-Ap layers, each of which had a different reporter gene: the cDNA of *firefly luciferase* (*FL*) or of *Renilla luciferase* (*RL*). Each DF-Ap layer was fabricated on a polystyrene substrate via a precursor-assisted biomimetic process using a simplified alternate dipping treatment [21,25]. The resulting substrates (termed Samples DF-FL and DF-RL, as listed in Table 1) with the DF-Ap layers were adjacently placed and used as scaffolds for culturing Chinese hamster ovary-K1 (CHO-K1) cells to allow area-specific dual gene transfer in the same well (Figure 1). We employed the CHO-K1 cell line, because it is well-characterized, easy to culture and transfect, and has been used in our previous studies [21,22,26,27]. The FL and RL activities of the cells cultured on each DF-Ap layer were evaluated by a dual luciferase assay system.

Second, we used the precursor-assisted biomimetic process to prepare another set of two DF-Ap layers, each of which had a different differentiation factor gene: the cDNA of the *vascular endothelial growth factor* (*VEGF*) [44] or of the *bone morphogenetic protein-2* (*BMP-2*) [45]. The resulting substrates (termed Samples DF-V and DF-B, as listed in Table 1) with the DF-Ap layers were then applied to preliminary dual gene transfer studies sing an experimental system that was similar to that illustrated in Figure 1 and multipotential C3H10T1/2 cells. The C3H10T1/2 cells are VEGF- and BMP-2-responsive mouse embryonic cells. VEGF and BMP-2 are vascular endothelial and osteogenic differentiation factors, respectively. The C3H10T1/2 cells cultured on each DF-Ap layer were assayed for *VEGF*, *BMP-2*, and differentiation marker gene expression by real-time PCR.

## 2. Results

### 2.1. Surface Structures of Samples

**Figure 2:**
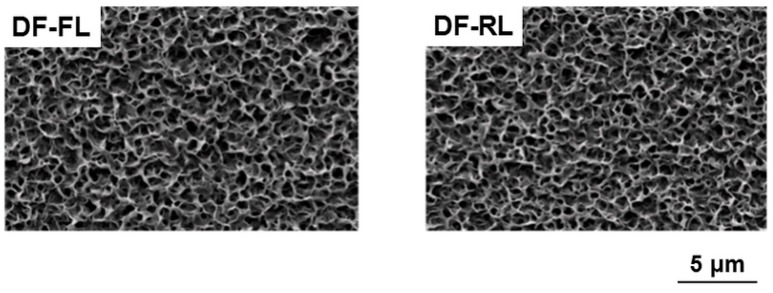
Scanning electron microscopy (SEM) images of the surfaces of the DNA–fibronectin–apatite composite layers (DF-Ap layers) immobilizing *firefly luciferase* (*FL*) gene (Sample DF-FL) and *Renilla luciferase* (*RL*) gene (Sample DF-RL).

**Figure 3:**
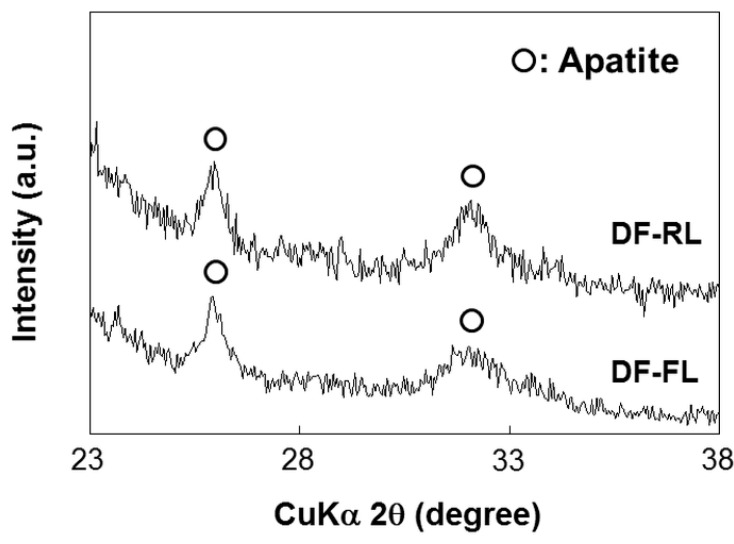
Thin-film X-ray diffraction (TF-XRD) patterns of the surfaces of the DNA–fibronectin–apatite composite layers (DF-Ap layers) immobilizing *firefly luciferase* (*FL*) gene (Sample DF-FL) and *Renilla luciferase* (*RL*) gene (Sample DF-RL).

**Figure 4:**
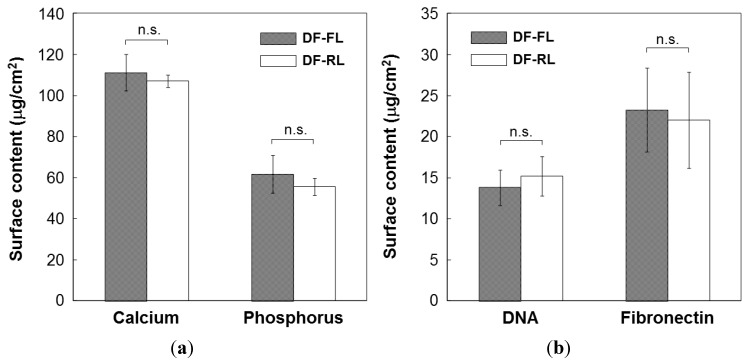
Contents of (**a**) calcium and phosphorus; and (**b**) DNA and fibronectin of the DNA–fibronectin–apatite composite layers (DF-Ap layers) immobilizing *firefly luciferase* (*FL*) gene (Sample DF-FL) and *Renilla luciferase* (*RL*) gene (Sample DF-RL). The results shown are the mean and standard deviation of three independent experiments.

The formation of DF-Ap layers on the surfaces of Samples DF-FL and DF-RL was confirmed by scanning electron microscopy (SEM), thin-film X-ray diffractometry (TF-XRD), and chemical analyses by inductively coupled plasma atomic emission spectrometry (ICP) and ultraviolet-visible spectrophotometry (UV-Vis). Uniform layers with a microscale cavernous and lumpy structure, as shown in the SEM images in Figure 2, were observed over the entire surface of both samples. No noticeable differences in surface morphologies were observed under SEM between these two layers. These layers were composed of low-crystalline apatite, as revealed by their TF-XRD patterns (Figure 3). The amount of apatite was comparable in Samples DF-FL and DF-RL because there was no significant difference in calcium and phosphorus contents on their surfaces (Figure 4a). The two apatite layers on Samples DF-FL and DF-RL immobilized plasmid DNA and fibronectin to a similar extent, according to the UV-Vis results (Figure 4b). These results indicate that the DF-Ap layers formed on Samples DF-FL and DF-RL were comparable in physicochemical properties, although the immobilized plasmid DNA was different (pGL3 in Sample DF-FL and pRL-TK in Sample DF-RL).

### 2.2. Cotransfer of Reporter Genes by Conventional Lipofection

To evaluate the trans effect of the promoters in the pGL-3 and pRL-TK vectors, a preliminary cotransfer study was performed using the conventional particle-mediated lipofection system. The mass percentage composition of pGL-3:pRL-TK was set at either 50:50 or 5:95, with a retention of a constant total DNA dose. When the percentage composition of pGL3:pRL-TK was 50:50 (in mass %), the cotransfected CHO-K1 cells exhibited the RL activity (4.6 × 10 and 3.3 × 10 counts in two independent experiments) that was four orders of magnitude lower compared with the FL activity (6.3 × 10^5^ and 5.8 × 10^5^ counts). When the percentage composition of pGL3:pRL-TK was changed from 50:50 to 5:95, the RL activity (4.5 × 10^4^ and 1.6 × 10^4^ counts) of the cells increased by three orders of magnitude and got closer to the FL activity (1.6 × 10^5^ and 2.5 × 10^5^ counts). At this percentage composition (pGL3:pRL-TK = 5:95), the dose of pRL-TK was approximately twenty times as much as that of pGL3. Despite this, the RL activity of the cells was still slightly lower than the FL activity.

### 2.3. Dual Reporter Gene Transfer Using a Pair of DF-Ap Layers

**Figure 5:**
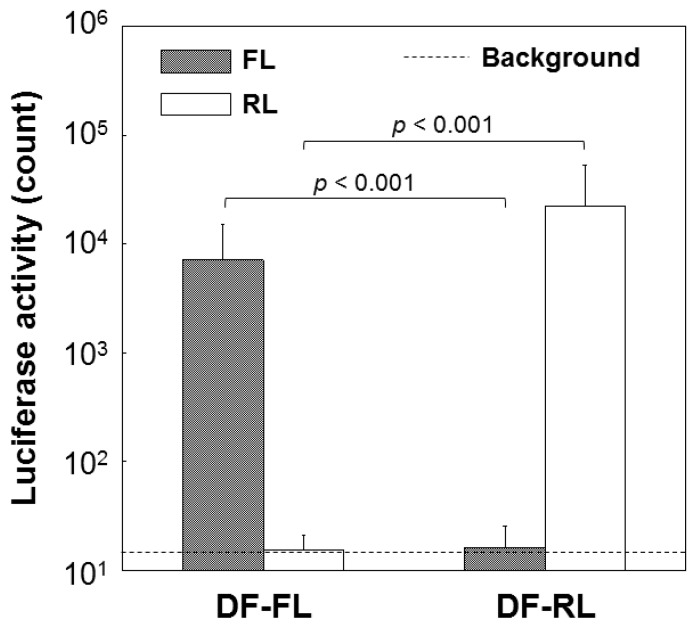
Luciferase (firefly luciferase (FL) and Renilla luciferase (RL)) activity in CHO-K1 cells cultured on the DNA–fibronectin–apatite composite layers (DF-Ap layers) immobilizing *FL* gene (Sample FL) and *RL* gene (Sample DF-RL). Both Samples FL and RL were adjacently placed in one well of a four-well chamber slide. The results shown are the mean and standard deviation of five independent experiments.

Area-specific dual gene transfer on the DF-Ap layers was successfully demonstrated using the *FL* and *RL* reporter genes. As shown in Figure 5, the FL activity of the CHO-K1 cells cultured on Sample DF-FL was approximately two orders of magnitude higher than that of the cells cultured on Sample DF-RL. On the other hand, the RL activity of the cells cultured on Sample DF-RL was approximately three orders of magnitude higher than that of the cells cultured on Sample DF-FL. The FL and RL activities of the cells cultured on Samples DF-RL and DF-FL, respectively, were both at the background level, *i.e.*, the luminescence level in the measurement without any test solution.

**Figure 6:**
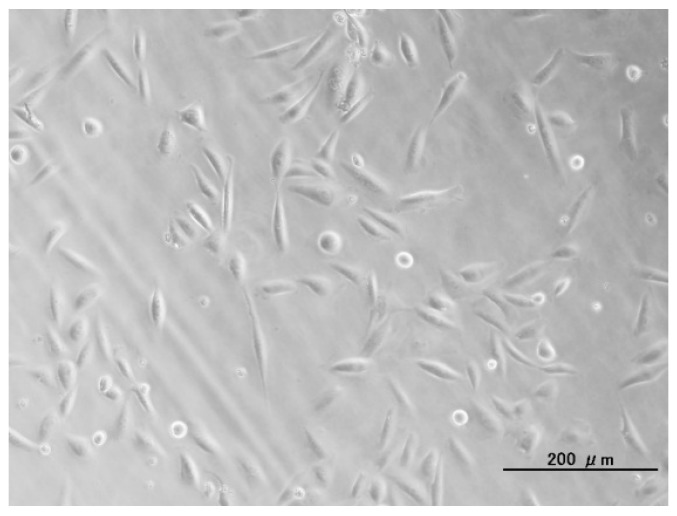
Optical microscopy image of CHO-K1 cells cultured on the DNA–fibronectin–apatite composite layer (DF-Ap layer) immobilizing *firefly luciferase* (*FL*) gene (Sample DF-FL). The scale bar indicates 200 μm.

Note that preparation conditions of the DF-Ap layers (to be described in Section 4.3) were decided to maximize the fibronectin content and gene transfer efficiency of the layer [21]. As shown in Figure 6, the CHO-K1 cells adhered well to the surface of the DF-Ap layer, most likely due to the cell adhesion activity of fibronectin immobilized in the DF-Ap layer [21].

### 2.4. Dual Differentiation Factor Gene Transfer Using a Pair of DF-Ap Layers

We performed preliminary dual gene transfer studies using the differentiation factor genes *VEGF* and *BMP-2* and multipotential C3H10T1/2 cells. Generally, gene transfer to multipotential cell lines like C3H10T1/2 is more difficult than that to easy-to-transfect cell lines like CHO-K1. Despite this, our gene transfer system using the DF-Ap layer was considered to be valid also for the C3H10T1/2 cells. Preliminary real-time PCR results of two independent experiments indicated a potential increase (2–4 orders of magnitudes) in *VEGF* expression level in the C3H10T1/2 cells cultured on Sample DF-V compared with that in the cells cultured on Samples DF-B and F (negative control) with a fibronectin–apatite composite layer. The real-time PCR results also suggested 2–4 orders of magnitudes higher *BMP-2* expression level in the C3H10T1/2 cells cultured on Sample DF-B compared with that in the cells cultured on Samples DF-V and F.

The real-time PCR results showed a sign of different expression patterns of differentiation marker genes in the C3H10T1/2 cells depending on the type of samples used for cell culturing: Samples F, DF-V, and DF-B. The results indicated a potential increase (3.3-fold in one experiment and 3.7-fold in the other experiment) in expression level of the endothelial differentiation marker *CD31* in the cells cultured on Sample DF-V than that in the cells cultured on Sample F. The cells cultured on Sample DF-B showed no sign of enhanced *CD31* expression level compared with the cells cultured on Sample F. On the other hand, the cells cultured on Sample DF-B showed a sign of enhanced expression level of osteogenic differentiation marker *alkaline phosphatase* (*ALP*) (3.4-fold in one experiment and 5.6-fold in the other experiment) compared with the cells cultured on Sample F. These preliminary results using the differentiation factor genes support the results of dual gene transfer studies using the *FL* and *RL* reporter genes (Section 2.3).

## 3. Discussion

Four kinds of DF-Ap layers were fabricated using the *FL* and *RL* reporter genes and the *VEGF* and *BMP-2* differentiation factor genes (Table 1). According to SEM (Figure 2), TF-XRD (Figure 3), ICP (Figure 4a), and UV-Vis (Figure 4b) results, these layers were similar in their surface morphology, crystalline phase, and the contents of apatite, plasmid DNA, and fibronectin, all of which are controlling factors that affect gene transfer efficiency [21,25,46]. The critical difference among these DF-Ap layers lies only on the type of plasmid DNA that was immobilized within the layer. Therefore, the effect of the physiochemical factors of the DF-Ap layers on gene expression efficiency was possibly negligible in the subsequent gene transfer studies.

The dual gene transfer studies indicated that the DF-Ap layers have the potential to mediate area-specific dual gene transfer and to stimulate cells on an area-by-area basis, even in the same well. As shown in Figure 5, the CHO-K1 cells cultured on the pGL3-derived DF-Ap layer showed only FL activity. In contrast, the cells cultured on the pRL-TK-derived DF-Ap layer exhibited only RL activity. The cotransfer of the *FL* and *RL* genes into the cells was unlikely to occur, taking into account the performance of the pGL3 and pRL-TK vectors, which was compared by the preliminary cotransfer study (Section 2.2). In the preliminary cotransfer study using conventional lipofection, the cotransfected CHO-K1 cells exhibited a slightly higher FL activity than RL activity, even when the dose of pGL3 was only approximately one twentieth that of pRL-TK (pGL3:pRL-TK = 5:95). This may be attributed to the trans effect [47] between the promoters/enhancers present in pGL3 and pRL-TK, *i.e.*, the promoters and enhancers in pGL3, which included the *FL* gene, were stronger than those in pRL-TK, which included the *RL* gene. Therefore, even if only a small amount (e.g., one twentieth of *RL*) of the *FL* gene from the pGL3-derived DF-Ap layer (on Sample DF-FL) was cotransferred into the cells on the pRL-TK-derived DF-Ap layer (on Sample DF-RL), those cells should have exhibited not only *RL* but also *FL* gene expression. Experimental results showed that this is not the case; the cells on the pRL-TK-derived DF-Ap layer exhibited only RL activity (Figure 5). From the results described above, cross-transfection from the two neighboring DF-Ap layers is likely to be denied. It is suggested that the plasmid DNA immobilized in the DF-Ap layer is locally transferred to the cells that adhered to the layer and hardly to the neighboring cells that were not in contact with this layer, as reported in our preliminary study [21].

The area-specific dual gene transfer on the DF-Ap layers was supported by the preliminary dual differentiation factor gene transfer study. As described in Section 2.4., the C3H10T1/2 cells cultured on Samples DF-V and DF-B were likely to proliferate into the cells with different gene expression patterns depending on the differentiation factor genes (*VEGF* and *BMP-2*) that were immobilized in the DF-Ap layer, even in the same well. The preliminary real-time PCR results suggested the enhanced *CD31* expression level in the C3H10T1/2 cells on the DF-Ap layer immobilizing *VEGF* gene (Sample DF-V) than in the cells on the fibronectin–apatite composite layer (Sample F). The results also suggested the enhanced *ALP* expression level in the cells on the DF-Ap layer immobilizing *BMP-2* gene (Sample DF-B) than in the cells on the fibronectin–apatite composite layer (Sample F). The expression of these differentiation markers (*CD31*, *ALP*) in the cells may be stimulated because of the enhanced expression of the differentiation factor genes (*VEGF*, *BMP-2*). Note that there seemed to be no noticeable difference in *CD31* expression level between the cells cultured on the DF-Ap layer immobilizing *BMP-2* gene (Sample DF-B) and the cells cultured on the fibronectin–apatite composite layer (Sample F). This could be caused by the area-specific dual gene transfer on the DF-Ap layers, as demonstrated in Figure 5. Although more detailed analyses are required in the future, these preliminary results suggest that, as a consequence of dual gene transfer, the C3H10T1/2 cells may have the potential to change into a different type of cells on Samples DF-V and Sample DF-B.

Considering the efficiency of our gene transfer system, approximately 7 μg of plasmid DNA was immobilized within the DF-Ap layer on a single substrate (10 mm × 5 mm) (Figure 4b) and transferred to the cells, resulting in approximately 10^4^ counts in the luciferase assay (Figure 5). In the conventional particle-mediated lipofection system, 0.4 μg of plasmid DNA was used, resulting in approximately 10^6^ counts in the luciferase assay (data not shown). These findings suggest that the efficiency of our gene transfer system using the DF-Ap layer is not as high as the conventional lipofection system. However, our gene transfer system is valid in the presence of serum, provides a biocompatible surface [18] with good cell adhesion property (Figure 6), allows slow and sustained release of DNA [20,22,27], and is capable of stimulating cells on an area-by-area basis as described in the following paragraph, which would be advantages in tissue engineering applications.

In the present study, the cells were cultured on a pair of DF-Ap layers together in the same medium in the same well, and these layers were placed next to each other at a very narrow distance (approximately 100 μm) (Figure 1). In such situations, area-specific dual gene transfer could be difficult when using conventional particle-mediated systems because of the diffusion of DNA complexes in aqueous media. Therefore, the surface-mediated system using the DF-Ap layers is advantageous over conventional particle-mediated systems in terms of the spatial control of gene transfer. According to our previous results, area-specific gene transfer is possible not only with the DF-Ap layers but also with other CaP-based composite layers [26]. Moreover, CaP-based composite layers can be fabricated on various substrate materials using the precursor-assisted biomimetic process [25]. On the other hand, CaP-based composite layers can be fabricated on an intended area/position of substrate materials using area-specific CaP coating techniques (e.g., laser-assisted biomimetic process [48,49]). In combination with such an area-specific coating technique, multiple CaP-based composite layers that immobilize different genes could be patterned on a scaffold surface. Using an appropriate design of scaffolds and choice of genes for immobilization, it may be possible to produce the intended cell type at an intended area/position on a scaffold, and eventually, highly structured tissues that comprise different types of cells. However, this is a challenge that will be addressed in a future study.

## 4. Materials and Methods

### 4.1. Materials

Four types of plasmid DNAs (0.7‒1.2 mg/mL) were prepared from four different genes (Table 1). A pGL3-Control vector including the cDNA of *FL* (Promega Corporation, Fitchburg, WI, USA) and a pRL-TK vector including the cDNA of *RL* (Promega) were used in the reporter gene transfer studies. The pGL3-Control vector contains the SV40 promoter/enhancer, whereas the pRL-TK vector includes the herpes simplex virus (HSV) promoter. The pCI-neo vector (Promega) with the human cytomegalovirus (CMV) enhancer/promoter was employed in the cell differentiation study using the *VEGF* and *BMP-2* genes. The cDNA of human *VEGF* and that of human *BMP-2* were inserted into the multiple cloning site of pCI-neo using the SalI and NotI sites at the linker ends (the resulting plasmid DNAs are termed pCI-*VEGF* and pCI-*BMP*, respectively). The cDNAs of *VEGF* and *BMP-2* were cloned from HeLa cells by reverse transcription PCR. The cDNA of *VEGF* was amplified using the following primers: forward primer, 5ꞌ-AGAGTCGACCTACCTCCACCATGCCAAGT-3ꞌ and reverse primer, 5ꞌ-ACTGCGGCCGCTGGTGAGAGATCTGGTTCCC-3ꞌ. The cDNA of *BMP-2* was amplified using the following primers: forward primer, 5ꞌ-AGAGTCGACTGAGCCTTTCCAGCAAGTTT-3ꞌ and reverse primer, 5ꞌ-ACTGCGGCCGCGGAACGTGTGTGTGTGGTGT-3ꞌ.

Polystyrene plates with 1-mm thickness were prepared by hot pressing polystyrene pellets (Sigma-Aldrich, St. Louis, MO, USA). The polystyrene plates were cut into 10 mm × 5 mm rectangular substrates, polished on both sides with a polishing compound (Tamiya polishing compound (Finish), Tamiya, Japan), washed ultrasonically with ethanol, and dried in a vacuum at 100 °C for 24 h. Fibronectin (1 mg/mL) from bovine plasma was purchased from Sigma-Aldrich, USA. Chemical reagents other than those listed above for the DF-Ap layer preparation were purchased from Nacalai Tesque Inc., Kyoto, Japan.

The CHO-K1 ovary cells (RIKEN BioResource Center, Tsukuba, Japan) and C3H10T1/2 embryonic cells (RIKEN BioResource Center) were used in the dual reporter gene and differentiation factor gene transfer studies, respectively (Table 1). RPMI1640 (Life Technologies Corporation, Carlsbad, CA, USA) and BME medium (Life Technologies Corporation) were used as the culture media for the CHO-K1 and C3H10T1/2 cells, respectively. Both culture media were supplemented with fetal bovine serum (Life Technologies Corporation) at a concentration of 10%.

### 4.2. Alternate Dipping Treatment for Precoating with the Apatite Precursor

The polystyrene substrate was subjected to oxygen plasma and subsequently to alternate dipping treatments to precoat with amorphous calcium phosphate (ACP), which is an apatite precursor [50]. The plasma treatment was performed in O_2_ gas using a compact ion etcher (FA-1, SAMCO Inc., Kyoto, Japan) at a pressure of 30 Pa and an energy density of 0.5 W/cm^2^ under an electric field operating at 13.56 MHz for 30 s [50]. The plasma-treated polystyrene substrate was alternately dipped in calcium and phosphate ion solutions, as described elsewhere [50,51]. In brief, the substrate was dipped in 20 mL of a 50:50 (in vol %) mixture of ethanol and 200 mM CaCl_2_ for 10 s, then dipped in 20 mL of 50 vol % aqueous ethanol for 1 s, and dried in air for a few minutes. The substrate was subsequently dipped in 20 mL of a 50:50 (in vol %) mixture of ethanol and 200 mM K_2_HPO_4_·3H_2_O for 10 s, dipped again in 20 mL of 50 vol % aqueous ethanol for 1 s, and dried in air for a few minutes. These alternate dipping operations in calcium and phosphate ion solutions were performed three times. The ACP-precoated polystyrene substrate was dried and then sterilized with ethylene oxide gas.

### 4.3. Biomimetic Process for Coating with DF-Ap Layers

Four kinds of coating solutions containing different plasmid DNAs (pGL-3, pRL-TK, pCI-VEGF, or pCI-BMP) and one control coating solution without plasmid DNA were prepared. First, a supersaturated CaP solution (142 mM NaCl, 1.50 mM K_2_HPO_4_·3H_2_O, 3.75 mM CaCl_2_, 50 mM tris(hydroxymethyl)aminomethane (Tris) [52]) was prepared by dissolving NaCl, K_2_HPO_4_·3H_2_O, HCl (40 mM), and CaCl_2_ in ultrapure water and then buffering the solution at pH = 7.40 at 25.0 °C with Tris and HCl [19,20,21,22,52]. Coating solutions were prepared by adding each plasmid DNA and fibronectin at a concentration of 40 and 10 μg/mL, respectively, to the CaP solution. The control coating solution was prepared by adding only fibronectin at a concentration of 10 μg/mL to the CaP solution. The plasmid DNA and fibronectin concentrations were determined according to our previous optimization results [21]. The coating solutions were sterilized by filtration.

The ACP-precoated polystyrene substrate (obtained in Section 4.2) was aseptically immersed in 1.5 mL of each coating solution at 25 °C for 24 h. After removal from the coating solution, the substrate was gently washed with ultrapure water before surface analysis (Section 4.4), or washed with phosphate-buffered saline (PBS) before cell culturing (Section 4.6, Section 4.7 and Section 4.8). The samples that were prepared using pGL3, pRL-TK, pCI-VEGF, and pCI-BMP plasmid DNAs were denoted as Samples DF-FL, DF-RL, DF-V, and DF-B, respectively (Table 1). The sample that was prepared using the control coating solution without plasmid DNA was denoted as Sample F. Sample F had a fibronectin–apatite composite layer on its surface [21], which was used as a negative control in the dual differentiation factor gene transfer study (Section 4.8).

### 4.4. Analysis of Sample Surfaces and Coating Solutions

The surface morphologies and structures of Samples DF-FL and DF-RL were examined by SEM (XL30, FEI Company Ltd., Hillsboro, OR, USA) and TF-XRD (RINT Ultima X, Rigaku Co., Tokyo, Japan) employing CuKα X-rays.

The coating solutions were clear and induced no spontaneous precipitation during the biomimetic process (24 h immersion of the substrate). Changes in the calcium and phosphorus concentrations and plasmid DNA and fibronectin concentrations of the coating solutions during the biomimetic process were quantified by ICP (Model PS 7800, Hitachi High-Tech Science Co., Tokyo, Japan) and UV-Vis (Model UV-2450; Shimadzu Corporation, Kyoto, Japan), respectively. To determine plasmid DNA concentration, the absorbance was measured at 260 nm. To assess fibronectin concentration, the absorbance was measured at 595 nm using a protein assay kit (Bio-Rad Laboratories, Inc., Hercules, CA, USA) based on the Bradford method. The content of calcium, phosphorus, plasmid DNA, and fibronectin immobilized on the sample surfaces was estimated by subtracting their final concentrations from the initial concentrations in the coating solution. Three substrates were used for each type of sample to obtain a mean value and a standard deviation. The data were compared using Student’s *t*-test, where the significance level was set at *p* < 0.05 for each analysis.

### 4.5. Cotransfer of Reporter Genes by Conventional Lipofection

Cotransfection was conducted using the commercial lipid transfection reagent Lipofectamine (Life Technologies Corporation), in accordance with the manufacturer’s recommended optimum conditions. First, the CHO-K1 cells were seeded in each well of a 24-well cell culture plate at a cell concentration of 5 × 10^4^ cells/0.5 mL/well. After culturing for 24 h in RPMI1640 medium with serum, the medium was replaced with 0.2 mL of serum-free Opti-MEM (Life Technologies Corporation) and then supplemented with 0.2 mL of serum-free Opti-MEM containing 0.4 μg of plasmid DNA (pGL-3 and pRL-TK) and 4 μL of the lipid transfection reagent. The mass percentage composition of pGL-3:pRL-TK was set at either 50:50 or 5:95, with a retention of a constant total DNA dose. After culturing for 5 h, the medium was replaced again with a serum-containing RPMI1640 medium. After culturing for another 43 h, the cells were washed three times with PBS and lysed in 200 μL of Passive Lysis Buffer (Promega). After vortexing, the cell lysate was centrifuged at 13,370× *g* for 2 min. The supernatant was analyzed using a dual luciferase assay kit (Promega) and a luminometer (Gene Light 55, Micro-tec Co., Ltd., Chiba, Japan) to evaluate the FL and RL activities of the cells.

### 4.6. Observation of Cells on DF-Ap Layer

Sample DF-FL was placed in one well of a twenty-four-well culture plate. The CHO-K1 cells were seeded on the sample at a cell concentration of 2.5 × 10^4^ cells/0.5 mL/well. After culturing for 48 h, the cells on the sample surface were observed by transmission optical microscopy (IX71, Olympus Co., Tokyo, Japan).

### 4.7. Dual Reporter Gene Transfer Using a Pair of DF-Ap Layers

As shown in Figure 1, Samples DF-FL and DF-RL were adjacently placed in one well of a four-well chamber slide (Nunc™ Lab-Tek™ II Chamber Slide™ System). The CHO-K1 cells were seeded on the samples at a cell concentration of 5 × 10^4^ cells/0.5 mL/well. After culturing for 72 h, the cells on each sample were assayed for FL and RL activities using the method described in the preceding section. Five substrates were used for each type of sample to obtain a mean value and a standard deviation. The data were compared using Student’s *t*-test.

### 4.8. Dual Differentiation Factor Gene Transfer Using a Pair of DF-Ap Layers

Samples DF-V and DF-B were adjacently placed in one well of the four-well chamber slide in the same manner as that illustrated in Figure 1. Sample F was placed in the well of the four-well chamber slide alone as a negative control. The C3H10T1/2 cells were seeded on the samples at a cell concentration of 5 × 10^3^ cells/0.5 mL/well and cultured for 14 days (the spent medium was replaced at 7 days of culture). After 14 days of culture, *VEGF*, *BMP-2*, *CD31*, and *ALP* gene expression was evaluated by real-time PCR (Mini Opticon real-time PCR system, Bio-Rad Laboratories Inc.). The cells cultured on each sample were washed three times with PBS, lysed, and centrifuged to obtain the supernatant. Total RNA was extracted from the supernatant using an RNA extraction kit (QIAGEN, Hilden, Germany). Five microgram of total RNA was reverse transcribed in a buffer containing 1 μL of oligo-dT primers (50 μM), 1 μL of deoxynucleotides (10 mM each), 20 U of RNase inhibitor (Takara Bio Inc., Shiga, Japan), and 200 U of PrimeScript^®^ RTase (Takara Bio). This mixture was incubated for 45 min at 42 °C and for 5 min at 95 °C. *VEGF*, *BMP-2*, *CD31*, *ALP*, and *β-actin* gene expression levels were detected using the following primers: forward primer 5ꞌ-AAGGAGGAGGGCAGAATCAT-3ꞌ and reverse primer 5ꞌ-ATGTTGGACTCCTCAGTGGG-3ꞌ for human *VEGF*; forward primer 5ꞌ-AAGGCACCCTTTGTATGTGG-3ꞌ and reverse primer 5ꞌ-CATGCCTTAGGGATTTTGGA-3ꞌ for human *BMP-2*; forward primer 5ꞌ-GCCCAATCACGTTTCAGTTT-3ꞌ and reverse primer 5ꞌ-GGCTTCCACACTAGGCTCAG-3ꞌ for mouse *CD31*; forward primer 5ꞌ-GAGCAGGAACACAAGTTTGC-3ꞌ and reverse primer 5ꞌ-GTTGCAGGGTCTGGAGAGTA-3ꞌ for mouse *ALP*; and forward primer 5ꞌ-GGACCTGGCTGGCCGGGACC-3ꞌ and reverse primer 5ꞌ-GCGGTGCACGATGGAGGGGC-3ꞌ for mouse *β-actin*. Each primer (12.5 pM) was added to a test solution containing 12.5 μL of iQ SYBR green supermix (Bio-Rad Laboratories) together with 0.5 μL of template sample (final volume, 25 μL). The gene expression levels were expressed as the delta–delta cycle time [Δ−Δ*C*(t)], which was normalized to the *β-actin* expression level. Two substrates were used for each type of sample in the real-time PCR assay.

## 5. Conclusions

We successfully demonstrated that the DF-Ap layers can mediate area-specific dual gene transfer. The cells cultured on two different DF-Ap layers exhibited different gene expression patterns depending on the gene that was immobilized in the underlying layer, even in the same well. DF-Ap layers with area-specific cell stimulation capability could be useful in tissue engineering applications.
